# PseudoMLSA: a database for multigenic sequence analysis of *Pseudomonas *species

**DOI:** 10.1186/1471-2180-10-118

**Published:** 2010-04-21

**Authors:** Antoni Bennasar, Magdalena Mulet, Jorge Lalucat, Elena García-Valdés

**Affiliations:** 1 Departament de Biologia, Microbiologia, Universitat de les Illes Balears, Campus UIB, 07122 Palma de Mallorca, Spain; 2 Institut Mediterrani d'Estudis Avançats (IMEDEA CSIC-UIB), Campus UIB, 07122 Palma de Mallorca, Spain; 3 Institut Universitari d'Investigació en Ciències de la Salut (IUNICS-UIB), Campus UIB, 07122 Palma de Mallorca, Spain

## Abstract

**Background:**

The genus *Pseudomonas *comprises more than 100 species of environmental, clinical, agricultural, and biotechnological interest. Although, the recommended method for discriminating bacterial species is DNA-DNA hybridisation, alternative techniques based on multigenic sequence analysis are becoming a common practice in bacterial species discrimination studies. Since there is not a general criterion for determining which genes are more useful for species resolution; the number of strains and genes analysed is increasing continuously. As a result, sequences of different genes are dispersed throughout several databases. This sequence information needs to be collected in a common database, in order to be useful for future identification-based projects.

**Description:**

The PseudoMLSA Database is a comprehensive database of multiple gene sequences from strains of *Pseudomonas *species. The core of the database is composed of selected gene sequences from all *Pseudomonas *type strains validly assigned to the genus through 2008. The database is aimed to be useful for MultiLocus Sequence Analysis (MLSA) procedures, for the identification and characterisation of any *Pseudomonas *bacterial isolate. The sequences are available for download via a direct connection to the National Center for Biotechnology Information (NCBI). Additionally, the database includes an online BLAST interface for flexible nucleotide queries and similarity searches with the user's datasets, and provides a user-friendly output for easily parsing, navigating, and analysing BLAST results.

**Conclusions:**

The PseudoMLSA database amasses strains and sequence information of validly described *Pseudomonas *species, and allows free querying of the database via a user-friendly, web-based interface available at http://www.uib.es/microbiologiaBD/Welcome.html. The web-based platform enables easy retrieval at strain or gene sequence information level; including references to published peer-reviewed articles, and direct external links to more specialized strain information databases (StrainInfo) and GeneBank (NCBI). The PseudoMLSA is intended to provide helpful strain-sequence information for a better and more comprehensive discriminative multigenic sequence based analysis of this special group of bacteria, contributing to enhance our understanding of the evolution of *Pseudomonas *species.

## Background

The genus *Pseudomonas *includes many species of environmental, clinical, agricultural, and biotechnological interest [1]. *Pseudomonas *is a large genus, currently comprised of more than 100 species that are phenotypically and genotypically well defined. Furthermore, new species are continuously being added to the genus, while others have been reclassified as *Burkholderia*, *Ralstonia, Comamonas, Acidovorax, Hydrogenophaga*, etc. The species currently classified as *Pseudomonas *have been compiled in a taxonomical web database [2].

Besides the phylogenetic, phenotypic, chemotaxonomical and serotyping descriptions, the recommended method for discriminating bacterial species is DNA-DNA hybridisation [3]. However, this method has limitations (it is time consuming, needs experience, does not define distances between species, and is not cumulative). In contrast, the MultiLocus Sequence Analysis (MLSA) is a rapid and robust classification method for the genotypic characterisation of a more diverse group of prokaryotes (including entire genera) using the sequences of multiple protein-coding genes [4]. In fact, Gevers and Coenye [5] have stated that multigenic sequence analysis, or MLSA, is starting to become a common practice in taxonomic studies, and in the future it may replace DNA-DNA hybridisations for bacterial species discrimination.

The principal aim of the MLSA procedure is the characterisation of bacterial isolates by combining the information contained in the sequences of several specific genes. The ideal, though probably unfeasible, approach for the classification of microorganisms based on MLSA would rely on the selection of a universal set of genes that permits the hierarchical classification of all prokaryotes [4,6]. However, genes that can be perfectly informative within a given genus or family may not be useful or even present in other taxa. For this reason, a more viable approach for microorganism classification schemes based on MLSA would be to design different gene sets useful for strains within a particular group, genus, or even family. Currently, each researcher selects specific genes that are not commonly used for other species; indeed, different genes are often selected for the same species. There is not a general criterion for determining which genes are more useful for taxonomic purposes [5]. As a result, sequences of different genes have been scattered throughout several databases. In order for this sequence information to be useful for future MLSA identification-based projects, it needs to be collected in a common database.

In many cases, the 16S rRNA gene sequence is not sufficiently discriminative for taxonomic purposes [7-9]. Consequently, several attempts have been made to identify other genes that can be used to determine the relatedness between genera or species. For example, the high rate of evolution of *gyrB *(gyrase subunit B) makes this gene valuable when discrimination within and between genera is needed. In the genus *Pseudomonas*, several other genes, *ampC, citS, flicC, oriC, oprI, *and *pilA*, from 19 environmental and clinical *Pseudomonas aeruginosa *isolates were analysed [10]. The 16S rRNA and *oprF *genes were also compared in 41 isolates of *Pseudomonas fluorescens *from clinical and environmental origin [11]. The *gacA *and *rpoB *genes were selected by de Souza [12] and Tayeb [8] to be analysed for the genus *Pseudomonas*. Yamamoto and Harayama [13] initially worked with 20 strains of *P. putida*, and 2 genes (*gyrB *and *rpoD*) were analysed and compared with 16S rRNA gene sequences of the same species. These authors later extended the study to other species of the genus *Pseudomonas*. The analysis of 125 strains of 31 species permitted the discrimination of complexes in the genus *Pseudomonas *[9]. Other authors showed an improved resolution in the phylogenetic relationships among *Pseudomonas *species by the combined analysis of several genes, such as *atpD*, *carA*, *recA*, and 16S rDNA, and new clusters were defined in the genus *Pseudomonas *[14]. The number of genes analysed is increasing, as is the case for the analysis of 10 genes in 58 *Pseudomonas *strains that generated 280 new entries in databases [15].

The possibility of Whole Genome Sequencing (WGS) represents a revolution for evolutionary and taxonomic analysis. Seventeen strains in the genus *Pseudomonas *have already been sequenced. For large bacterial populations the epidemiological or ecological approaches need simple methodological routines for which MLSA will remain a highly efficient and inexpensive technique useful for identifying species and strains relationships. Evidently, at least for sometime more than one technique will coexist.

Some facts are emerging from these recent analyses. The number of strains and genes analysed is increasing continuously, and the strains analysed are not solely bacterial pathogens. The number of genes that should be analysed does not need to be the same for identification purposes, depending on the genetic diversity of each group. The initial recommendation for typing clinical isolates was seven genes. The *ad hoc *committee for the re-evaluation of the species definition proposed a minimum of five housekeeping genes to achieve an adequately informative level of phylogenetic data [3]. *P. stutzeri *is a well studied example of a highly diverse species, and six genes were initially chosen to define the existing genomovars [16], but this number was later reduced to three: *gyrB*, *rpoD*, and 16S rDNA [17]. The usefulness of these genes in clarifying taxonomical descriptions has been demonstrated for *Pseudomonas *strain OX1 [18] and for the proposal of *P. chloritidismutans *as a junior name of *P. stutzeri *genomovar 3 [19].

Currently, the sequence data that have been generated for several genes are dispersed in databases, and the compilation of all these data is, while not difficult, labour intensive. However, a secondary database for MLSA is needed, one that is more specific and focused on *Pseudomonas *type strains to facilitate the species identification of *Pseudomonas *isolates. A good example is the recently available website called "EzTaxon" [20]. This website contains 16S rRNA gene sequences from all prokaryotic type strains, and represents an attempt to make the routine identification of isolates less time consuming. The compilation of an updated forum for the well-characterised (both phenotypically and genotypically) strains of *Pseudomonas *and for all of the genes analysed from these strains is the main objective of the new PseudoMLSA database.

## Construction and content

The PseudoMLSA database runs on a Mac OS X platform (version 10.4.11) with the Apache web server version 1.3.41 (Darwin), MySQL server (version 5.1.34) and PHP (version 5.2.4). The web server and all parts of the database are hosted at the Microbiology Area of the Biology Department of the Universitat de les Illes Balears (UIB), Spain. We have used the generic relational BioSQL model [21] to support and develop a shared database schema for storing sequence data, features, and annotation in a way that is interoperable between the BioPerl, BioPython, and BioJava projects. We have used MySQL as a supported Relational Database Management System (RDBMS), plus the associated python library. GenBank files are used to supply and maintain the information necessary for the database. The sequences, features, and annotations are introduced into the database using BioPython based scripts [22] and the SeqIO module [23]. The web interfaces that allow access the information available in the database online were written in the PHP programming language. The PseudoMLSA database includes tables of taxonomic information (strains, *Pseudomonas *validated species names, strain equivalencies) that are routinely updated. Finally, several interfaces for *in silico *molecular biology services were implemented for post-processing available sequence data. The installed programs include BLAST [24], a CLUSTAL W Multiple Sequence Alignments form [25] and the programs for phylogenetic inference included in the PHYLIP package [26].

## Utility and Discussion

The aims of this database project are: 1) maintenance of a well-described *Pseudomonas *type and strain collection, 2) construction of a sequence-based database of selected genes of members of the genus, and 3) implementation of analytical bioinformatics tools for the multi-sequence-based identification of *Pseudomonas *species. The database presented here and named PseudoMLSA, consists of more than 1,000 sequence entries from 99 *Pseudomonas *species with validly published names of the taxa concerned. The database covers more than 400 different strain entries (including type strains for each species), with information on strain equivalencies when it exists, together with the accession numbers and other features for 146 different genes. The list of genes includes the *rrn *operon genes (the 16S rRNA and 23S rRNA genes, the internally transcribed spacer ITS1, and the tRNA-Ala and tRNA-Ile genes), housekeeping (*atpD*, *gyrB*, *recA*, *rpoB, rpoD*, etc.), and functional genes (*car*, *cat*, *nir*, *nor*, *nos*, etc.). The data from the species *Pseudomonas stutzeri *are overrepresented in the PseudoMLSA database. Our laboratory has studied this species extensively for more than 20 years, and a large number of sequences of multiple genes have been accumulated. Furthermore, the existence in *P. stutzeri *of 19 well characterised genomic groups, called genomovars [27], has been a valuable test data set for the routine characterisation of new isolates on the basis of sets of gene sequences.

**Figure 1:**
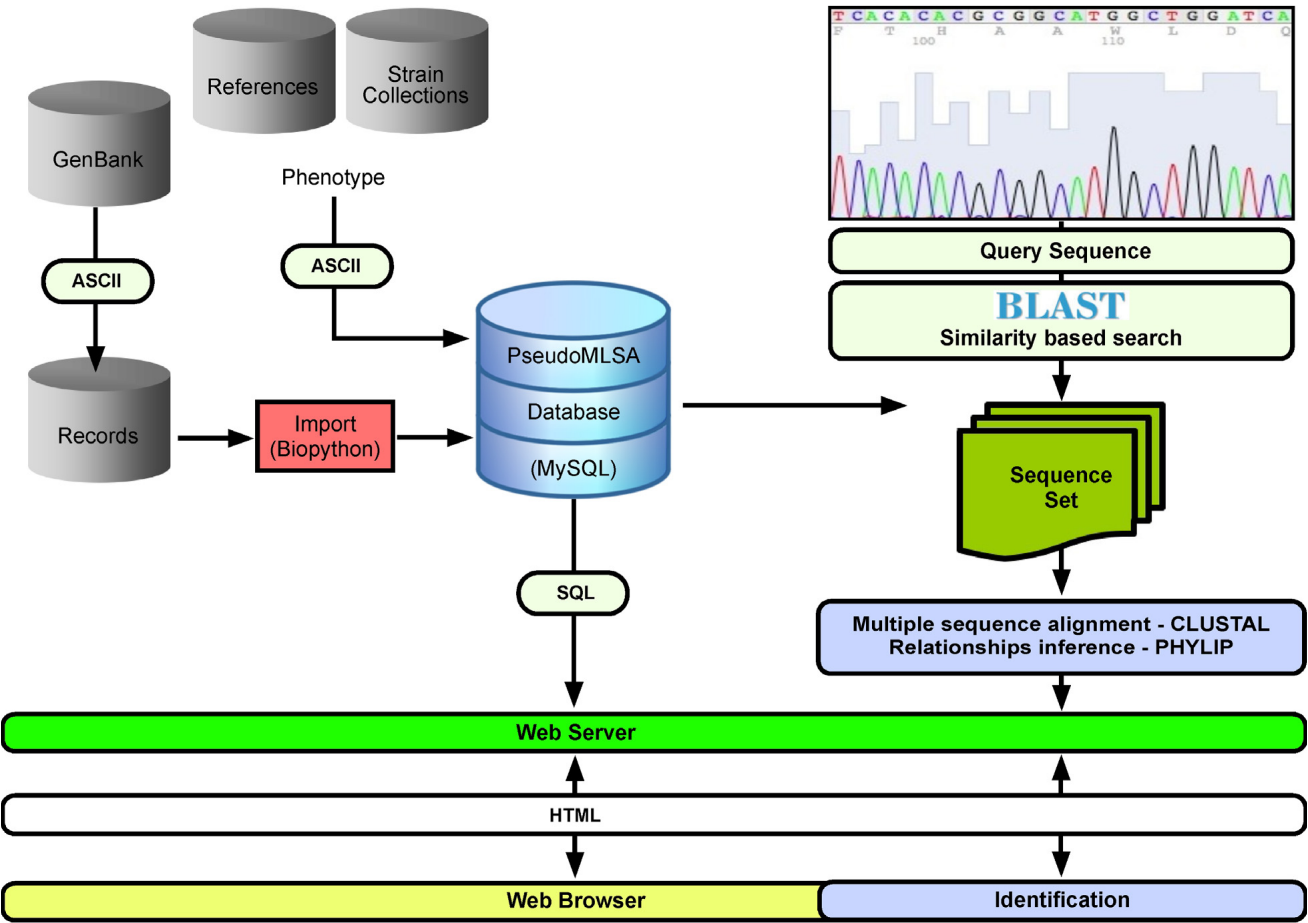
**General overview of the process for maintenance, queries, and analysis of gene sequences using the PseudoMLSA Database server http://www.uib.es/microbiologiaBD/Welcome.html**. The isolate, strain or *Pseudomonas *species information can be easily queried by searching against several fields. Furthermore, users can do sequence-based searches against database including user's own sequence datasets. Advanced searches are possible via configurable BLAST parameters. A more fine-tuned clustering analysis can be carried out with programs included in the PHYLIP package.

The implementation and data acquisition functions of the PseudoMLSA database are based on emerging standards for biological data [21,28], and therefore allow for the subsequent use of public routines (BioJava, BioPython and BioPerl). The database schema allows for several features, such as GenBank accession numbers, to be merged and stored as a single record (Figure 1). Gene sequences are obtained from primary databases like GenBank [29] and semi-automatically curated. Information for strains of *Pseudomonas *species is included in the databases from the GenBank report (data are imported through known accession numbers). In order to make use of validated information for *Pseudomonas *species and strains, the database schema includes tables that are responsible for updating and relating the validly described *Pseudomonas *species names with the corresponding synonymous names. Furthermore, additional database tables are maintained with the corresponding strain name equivalencies. Finally, all taxonomic names are maintained with, and linked out to, key taxonomic information sources like StrainInfo.net [30], a bioportal offering an integrated view of publicly available microbial cultures and their downstream information to facilitate the daunting task of tracking down an interesting strain of a given taxon. The StrainInfo.net bioportal [31] brings together the records of biological material kept at multiple biological resource centres into a single portal interface, with direct pointers to the relevant information at the collections' websites, providing both historical traces and geographical distribution of the strains they keep in culture. In addition, the information for *Pseudomonas *species and/or strains is automatically linked to related sequences in the public domain and refers to existing scientific publications that deal with the organism.

Since the alignment of nucleotide or amino acid sequences is one of the most important tools for researchers involved in gene sequence comparison for identification purposes, users can also upload their own sequence datasets to query against. The basic local alignment search tool (BLAST), which predominates as the fastest and most widely-used tool, has been included as a web-based interface to search against the PseudoMLSA sequence database. The BLAST program is widely used for sequence similarity searches [32] because it provides an easy way for a user to perform BLAST searches via a web server, and it suits the general purpose of searches against the curated PseudoMLSA database. Additionally, a web interface for PHYLIP programs [26,33] is implemented to carry out more precise evolutionary studies.

The PseudoMLSA database offers an interface for choosing between a user-definable set of target databases, and inputting user uploaded query sequences by pasting them directly into the query box, or by uploading sequences as FASTA files from a local computer. Users can also manipulate the BLAST parameters to glean more specific information. The results of a BLAST query to PseudoMLSA are presented with the typical BLAST report format which includes the query sequence name, subject sequence names, subject source database, bit score (linked to the pair-wise alignment result), percent identity, and E-values.

The number of gene sequences for strains in the genus *Pseudomonas *is continuously increasing, yet these sequences are scattered throughout existing databases. As a result, methods and databases are needed to integrate information from a variety of sources and to support faster and powerful analyses. In addition, in the specific case of the genus *Pseudomonas*, 16S rRNA gene sequence-based identification alone provides poor resolution due to the gene's slow evolution rate [8,34]. Moreover, the excess of sequences for non-type strains, together with the need for peer-reviewed databases of 16S rRNA gene sequences (routinely used for the identification of bacteria), creates discrepancies. The combined use of the 16S rRNA gene and other molecular sequences to analyse the phylogeny of *Pseudomonas *could provide a systematic approach to reduce such discrepancies. Achieving this goal requires building on the analysis initially conducted by the Yamamoto [9,13] and Tayeb [8] groups, who sequenced the genes *gyrB*, *rpoD *and *rpoB *respectively, and expanding it to include all known *Pseudomonas *species.

The PseudoMLSA Database server provides cumulative and reliable information to facilitate MultiLocus Sequence Analysis for studies of *Pseudomonas *taxonomy, phylogeny, and evolution. Furthermore, it serves as a reference repository for MLST, an unambiguous procedure for characterising isolates of bacterial species using the sequences of internal fragments of usually seven housekeeping genes. This method assigns as distinct alleles the different sequences present within a bacterial species and, for each isolate, the alleles at each loci define the allelic profile or sequence type [35]. Consequently, the information held in the PseudoMLSA database could play two essential roles in the field of *Pseudomonas *research: first, to fulfil the need for the integration of information about the genus *Pseudomonas *that is currently widely dispersed across existing databases; and second, as a platform for a consistent identification procedure based on the analysis of sets of multiple gene sequences to settle the difficulties in assigning new isolates to already existing *Pseudomonas *species, and for defining novel species.

## Conclusions

In summary, the relational database and the accompanying analysis utilities described here are necessary tools for integrating and linking sets of sequence information from different genes of the genus *Pseudomonas*, including universal genes with different rates of evolution (*rrn*, ITS, *gyrB*, *rpoD*), and specific genes for performing intra- and intergeneric comparisons on groups or species (for example, catecol-1,2-dioxigenase is characteristic of Palleroni's RNA homology group I of the genus *Pseudomonas *[1], or *nosZ *for denitrifying *Pseudomonas*). The PseudoMLSA Database is intended to provide reference sequences from strains, as well as *Pseudomonas *species information, both of which can be particularly helpful for MLSA of *Pseudomonas*. Ultimately, the purpose of the database is to afford a portable, accurate, and, highly discriminating system that can be used for the characterisation of *Pseudomonas *strains and isolates by nucleotide sequence based approaches via the internet.

## Availability and requirements

The PseudoMLSA database is freely accessible through a web-server at http://www.uib.es/microbiologiaBD/Welcome.html for searches for *Pseudomonas *strains and multigenic sequence-related information. The PseudoMLSA database is easily queried from this web interface and whithout special requirements. Researchers involved in the characterisation of *Pseudomonas *strains are invited to use the database, make suggestions and submit their sequences. All comments, queries, requests and corrections should be sent by email to toni.bennasar@uib.es. Furthermore, notifications for new entries in the GenBank for *Pseudomonas *gene sequences, will be gratefully acknowledged and should be sent to this address via email and accompanied by a reference to a published, peer-reviewed article. Users of PseudoMLSA are requested to cite this article when referencing the database. PseudoMLSA currently contains 1,297 entries of *Pseudomonas *gene sequences, but is expected to grow continuously thanks to the rapid development of MLSA and genome projects.

## Competing interests

The authors declare that they have no competing interests.

## Authors' contributions

AB designed the database and interface, performed the installation of required software, curated the database, and drafted the manuscript. MM helped to define the user requirements and prepared the strain, sequence, and reference data for the database. EGV conceived of the study and participated in its coordination. EGV interacted with AB to select and introduce the data. JL provided specialist knowledge on *Pseudomonas *taxonomy and phylogenetic analysis based on sequence data. JL and EGV equally oversaw the project. All authors helped to draft, read and approved the final manuscript.
